# Semi-quantitative analysis of visually normal ^123^I-FP-CIT across three large databases revealed no difference between control and patients

**DOI:** 10.1186/s13550-023-00983-6

**Published:** 2023-04-28

**Authors:** Anthime Flaus, Remi Philippe, Stephane Thobois, Marc Janier, Christian Scheiber

**Affiliations:** 1 Department of Nuclear Medicine, Hospices Civils de Lyon, Bron, Rhône, France; 2 Faculté de Médecine Lyon Est, Université Claude Bernard, Lyon 1, Lyon, France; 3 Lyon Neuroscience Research Center, INSERM U1028/CNRS UMR5292, Lyon, France; 4 Institut des Sciences, Cognitives Marc Jeannerod, UMR 5229, CNRS, CRNL, Université Claude Bernard, Lyon 1, Lyon, France; 5 Movement Disorder Clinic, Department of Neurology C, Pierre Wertheimer Neurological Hospital, Hospices Civils de Lyon, Bron, Rhône, France

**Keywords:** ^123^I-FP-CIT SPECT, Normal reference values, Age effect, Parkinsonism

## Abstract

**Background:**

To show the equivalence between the specific binding ratios (SBR) of visually normal ^123^I-FP-CIT SPECT scans from patients to those from healthy volunteers (Hv) or patients without dopaminergic degeneration to allow their use as a reference database.

**Methods:**

The SBR values of visually normal SPECT scans from 3 groups were studied: (1) suspected Parkinsonism and no diagnostic follow-up (ScanOnlyDB: *n* = 764, NM/CT 670 CZT, GE Healthcare), (2) no degenerative dopaminergic pathology after a 5-year follow-up (NoDG5YearsDB: *n* = 237, Symbia T2, Siemens Medical Solutions), and 3) Hv (HvDB: *n* = 118, commercial GE database). A general linear model (GLM) was constructed with caudate, putamen, and striatum SBR as the dependent variables, and age and gender as the independent variables. Following post-reconstruction harmonization of the data, DB were combined in pairs, ScanOnlyDB&NoDG5yearsDG and ScanOnlyDB&HvDB before performing GLM analysis. Additionally, ScanOnlyDB GLM estimates were compared to those published from Siemens commercial DB (SiemensDB) and ENC-DAT.

**Results:**

The dispersion parameters, *R*^2^ and the SBR coefficients of variation, did not differ between databases. For all volumes of interest and all databases, SBR decreased significantly with age (e.g., decrease per decade for the striatum: − 4.94% for ScanOnlyDB, − 4.65% for NoDG5YearsDB, − 5.69% for HvDB). There was a significant covariance between SBR and gender for ScanOnlyDB (*P* < 10^–5^) and NoDG5YearsDB (*P* < 10^–2^). The age-gender interaction was significant only for ScanOnlyDB (*P* < 10^–2^), and the p-value decreased to 10^–6^ after combining ScanOnlyDB with NoDG5YearsDB. ScanOnlyDB GLM estimates were not significantly different from those from SiemensDB or ENC-DAT except for age-gender interaction.

**Conclusion:**

SBR values distribution from visually normal scans were not different from the existing reference database, enabling this method to create a reference database by expert nuclear physicians. In addition, it showed a rarely described age-gender interaction related to its size. The proposed post-reconstruction harmonization method can also facilitate the use of semi-quantitative analysis.

**Supplementary Information:**

The online version contains supplementary material available at 10.1186/s13550-023-00983-6.

## Introduction

Iodine 123-radiolabeled 2β-carbomethoxy-3β-(4-iodophenyl)-N-(3-fluoropropyl) nortropane (or ^123^I-FP-CIT) scintigraphy is a biomarker of the presynaptic dopaminergic system that enables the assessment of the dopamine transporter availability. In case of suspected Parkinsonism, a ^123^I-FP-CIT single-photon emission computerized tomography (SPECT) examination [1] may be prescribed to rule out Parkinson’s disease in case of a visually normal (or grade ‘0’) scan [2]. Visual analysis remains the gold standard, but a semi-quantitative measurement of the specific binding ratio (SBR) can improve the inter- and intra-operator reproducibility and can help with the interpretation of equivocal scintigraphy scans [3–5]*.*

SBR analysis is based on a comparison with reference values corresponding to age-matched healthy volunteers (Hv) from large multicenter studies, such as the European Normal Control Database of DaTSCAN (ENC-DAT) [6], the Japanese multicenter database of healthy controls for ^123^I-FP-CIT SPECT [7], and the Parkinson Progression Marker Initiative (PPMI) [8]; GE Healthcare (Chicago, IL, USA) commercializes a database of Hv SBR values sampled from the latter [8] that accompanies the semi-quantitative analysis software DaTQUANT™. A potential limitation of the use of these databases is the additional instrumental variance in SBR values caused by the heterogeneity in equipment, despite the inter-center (inter-system) calibration, which could reduce their sensitivity [9, 10]*.* Alternatively, SBR reference values are obtained locally and retrospectively from normal ^123^I-FP-CIT routine examinations of a patient group presenting heterogeneous suspected diagnoses, but without dopaminergic neurodegenerative pathology, after a follow-up period lasting up to 5 years [11, 12].

SBR values depend on the imaging method, so a direct comparison between datasets acquired using two different imaging systems is not readily achievable. However, variations according to age or gender should be independent of the imaging chain and could be used as metrics for inter-database comparisons. Nevertheless, as attenuation correction methods have proved to influence such variations [13], database comparisons should be restricted to data using the same attenuation correction method. Databases have therefore been compared via estimates of the variables in general linear regression model analysis and by the proportion of the explained variance (*R*^2^) [11, 12]. No significant difference was observed between databases from Hv and those from patients without dopaminergic neurodegenerative pathology. However, these results are still debated, as SBR could also depend on comorbidities; recent study found that the mean posterior putamen SBR value was 8% higher in a group of symptomatic patients without dopaminergic neurodegenerative pathology than in a group of Hv [14].

We hypothesized that it is possible to generalize this absence of difference compared to Hv or patients without disease after follow-up to all normal routine scintigraphy scans of patients presenting suspected Parkinsonism, even without collecting follow-up information (ScanOnlyDB). Therefore, the objective of the present study was to show the absence of difference of the estimates and dispersion parameters from the normal routine scintigraphy SBR values of patients with suspected Parkinsonism to those of Hv or patients without dopaminergic degeneration.

## Patients and methods

### Description of ScanOnlyDB

Consecutive routine ^123^I-FP-CIT SPECT scans obtained from September, 2016 to March, 2020, at the Hospices Civils de Lyon, were reviewed and classified either as “normal” or “pathologic” through a visual analysis [2] blinded to the clinical data (CS-AF); only patients with a normal scan were included. The clinically suspected diagnoses before the scan are listed in Additional file 1: Table S1.

#### SPECT processing and SBR computations

Three hours after ^123^I-FP-CIT administration (mean ± standard deviation [SD] dose: 146 ± 24 MBq; Additional file 1: Table S1) acquisitions, including the whole head, were performed using an NM/CT CZT 670 (GE Healthcare, Chicago, IL, USA) camera equipped with a WEUH collimator. A total of 120 projections were acquired within 30 min. The distance to the axis of rotation was fixed at 14 cm. The photopeak imaging window was set to 159 keV (− 6%, + 5%). An X-ray computed tomography (CT) was performed (120 kV, noise index 6.5, 1.5-mm slice thickness).

The reconstruction protocol and the semi-quantitative analysis method were identical to those of the commercial HvDB database using DaTQUANT™ (GE Healthcare, Chicago, IL, USA). Two attenuation correction (AC) methods were used: 1) linear attenuation: Chang method with µ = 0.11 cm^−1^ (Chang AC) [15], and 2) the attenuation method based on the CT data (CT AC).

### Description of reference databases

1) The NoDG5YearsDB is composed of scans from patients presenting an indication for a ^123^I FP-CIT examination without degenerative dopaminergic pathology after a mean ± SD follow-up duration of 5 ± 1 years. It was acquired at the Hospices Civils de Lyon using a Symbia T2 SPECT-CT camera (Siemens Medical Solutions USA, Inc.) between January 2008 and December 2015, following European recommendations [1]. This cohort contained 237 subjects, matched for age and gender; their mean ± SD age was 62 ± 15 years, and 117 (49.4%) were women. The diagnoses are listed in Additional file 1: Table S1. This cohort included 25 (8.5%) young adults with attention deficit/hyperactivity disorder. Patients with suspected atypical Parkinsonism were excluded. SBRs were computed using an in-house semi-quantitation method (Additional file 2). Scientists from Siemens (Siemens Medical Solutions USA, Inc.) simultaneously and independently processed these raw data, using the Flash3D reconstruction method and CT AC. It resulted in different variants of corresponding reference SBR values made commercially available [12]. This database is hereafter referred to as SiemensDB.

2) The HvDB (DaTQUANT™, GE Healthcare, Chicago, IL,USA) was composed of visually normal scans from 118 Hv from the PPMI cohort [8]. The mean ± SD Hv age was 60 ± 13 years and 73 (61%) were men. Data were acquired in several centers using different gamma cameras.

A detailed description of NoDG5YearsDB and HvDB can be found in Additional file 3.

### Statistical analyses

**Fig. 1:**
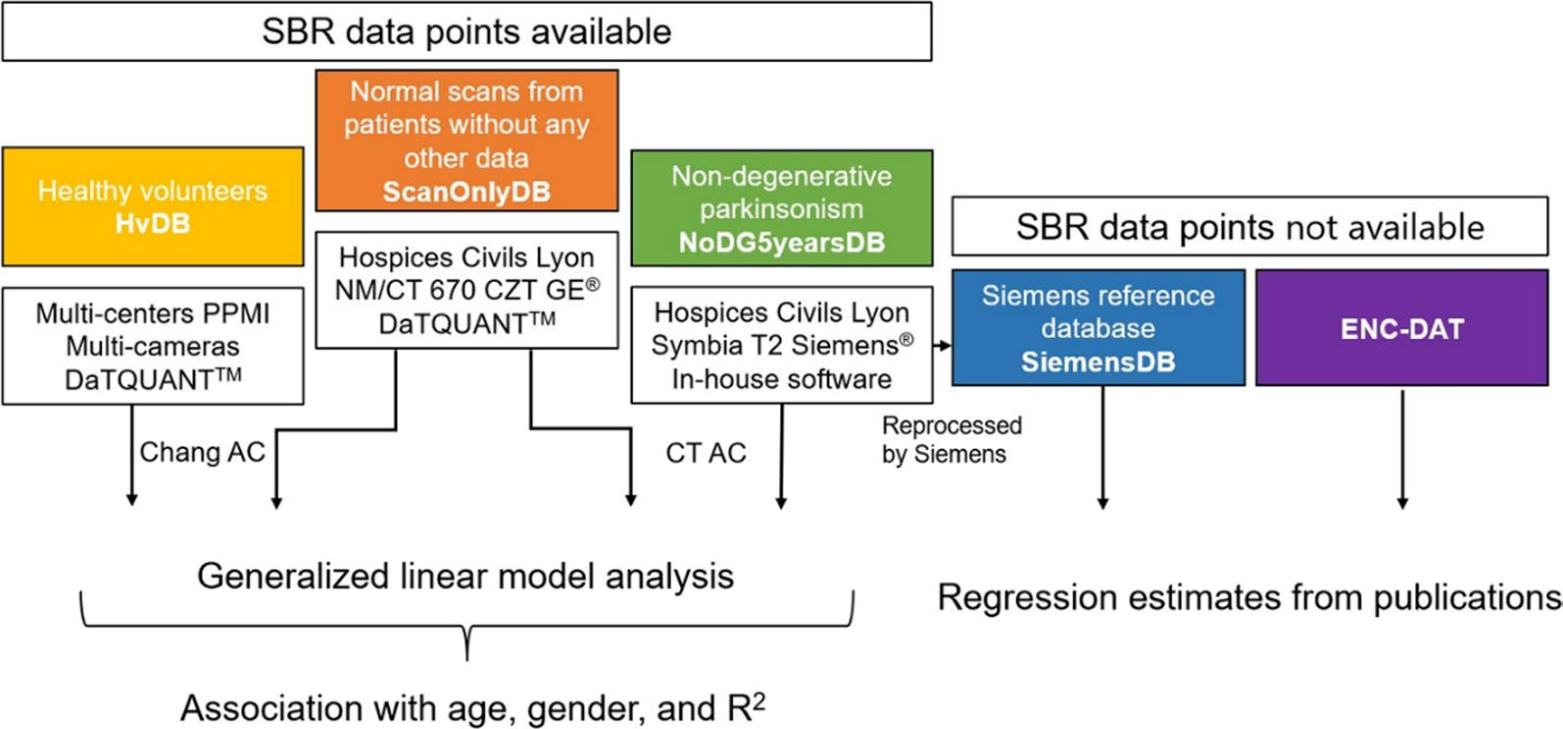
Schematic presentation of the analysis plan

HvDB: Healthy volunteer database; ScanOnlyDB: Visually normal scintigraphy database of patients with suspected Parkinsonism, blinded clinical information; NoDG5yearsDB: database of patients without dopaminergic degenerative pathology after a 5-year follow-up; ENC-DAT: European Normal Control Database of DaTSCAN™; Siemens reference database (SiemensDB); CT: computerized tomography.

GLMs (details in Additional file 4) allowed the estimation of the covariation of the striatum, the putamen, the caudate, and the putamen/caudate (P/C ratio) SBR with the tested independent variables: age, gender, and their interaction, for each database (Fig. 1, Left). The metrics used to estimate the dispersion of SBR values of the databases were the SD, coefficient of variation (CoV) after correction for mean age, and the *R*^2^ of the GLM. We used Cohen’s formula [16] to compare our correlation coefficients, obtained with the GLM, to those corresponding to SiemensDB and ENC-DAT [6, 12, 17] (Fig. 1, Right).

As SBR values depend on the acquisition and processing protocols, a post-reconstruction harmonization was necessary before GLM analysis of combined databases. First, each database was submitted to a GLM model testing for age dependency (intercept ± Standard Error [SE], slope ± SE). Then, as previously mentioned, due to the influence of the AC method on the parameters studied, we proceeded by pair according to the attenuation correction used. The signed intercept difference was applied to harmonize ScanOnlyDB AC CT with NoDG5yearsDB and ScanOnlyDB Chang AC with HvDB (value of the offset ~ 10% SBR). Details of database harmonization can be found in Additional file 5.

Statistical analyses were performed using Matlab 7 2021a (The MathWorks, Natick, MA, USA). Continuous variables were expressed as mean ± SD and compared using the Wilcoxon test or the Student's t-test, according to their distribution. Discrete variables were expressed as count (percentage) and compared using the Chi-squared test. Significance was set at *P* < 0.05.

## Results

### Characteristics of ScanOnlyDB

A total of 764 subjects matched for gender and age were included in the ScanOnlyDB; the descriptive statistics are summarized in Table 1. The main clinically suspected diagnoses before the ^123^I FP-CIT study of the included subjects were: essential tremor (*n* = 181, 23.7%), post-neuroleptic Parkinsonism (*n* = 177, 23.2%), atypical Parkinsonism (*n* = 95, 13.5%), and dementia without dopaminergic denervation (*n* = 103, 12.4%). For the complete list, see Additional file 1: Table S1. Briefly, the striatum, the caudate, and the putamen mean SBR values of women were 6.6%, 7.1%, and 8.4% higher, respectively, than those of men (*P* < 0.001). The caudate mean SBR value was higher than the putamen mean SBR value for both genders (*P* < 0.05). There was no significant asymmetry for the striatum as the mean ± SD SBR was 2.64 ± 0.50 for the left side and 2.62 ± 0.49 for the right side (*P* = 0.53). CT AC-corrected SBR values were lower than Chang AC-corrected SBR values for all striatal regions (*P* < 0.0001).

**Table 1:** Characteristics according to gender

Gender	Age (years)	Injected dose (MBq)	SBR_striatum_	SBR_caudate_	SBR_putamen_
Male (*n* = 397, 52%)	70.81 ± 10	146 ± 24	2.55 ± 0.46	2.93 ± 0.55	2.37 ± 0.45
Female (*n* = 367, 48%)	70.02 ± 11	146 ± 24	2.72 ± 0.52	3.12 ± 0.62	2.54 ± 0.51
*P* = 0.46	*P* = 0.58	*P* = 0.60	*P* < 0.00001	*P* < 0.0003	*P* < 0.0006

### Data from ScanOnlyDB are presented

Mean and standard deviation of age, injected dose, and Chang AC Specific Binding Ratio (SBR) values for the striatum, the caudate and the putamen, for the database composed of visually normal scans (suspected Parkinsonism and no diagnostic follow-up, ScanOnlyDB).

### ScanOnlyDB GLM with age as independent variable

The striatum, the caudate, and the putamen SBR decreased with age (*P* < 0.001; Table 2). For example, the mean ± SD SBR variation for the striatum (Chang AC) was − 4.94 ± 0.27% per decade. For the posterior putamen (CT AC) the estimated intercept ± SE was 3.21 ± 0.06% and slope 0.020 ± 0.0009. In addition, the SBR_putamen_ to SBR_caudate_ ratio indicated a clear covariance with age (*P* < 10^–5^).

**Table 2:** linear regression between Specific Binding Ratio according to attenuation correction methods and age for ScanOnlyDB

Volume of interest	Parameters	SBR Chang AC	SBR CT AC
		Estimate ± SE	Estimate ± SE
Striatum	Intercept	4.38 ± 0.074	3.61 ± 0.059
	Slope	− 0.025 ± 0.0010	− 0.020 ± 0.0008
Caudate	Intercept	4.79 ± 0.092	3.92 ± 0.075
	Slope	− 0.025 ± 0.0013	− 0.020 ± 0.001
Putamen	Intercept	4.18 ± 0.072	3.47 ± 0.058
	Slope	− 0.025 ± 0.001	0.020 ± 0.0008
Putamen/caudate	Intercept	0.914 ± 0.018	0.90 ± 0.018
	Slope	− 0.001 ± 0.0003	− 0.001 ± 0.0003

Linear regression parameters derived from Specific Binding Ratio (SBR)of visually normal scans (suspected Parkinsonism and no diagnostic follow-up, ScanOnlyDB).

Chang AC: a numerical method for attenuation correction, CT AC: computed tomography-based attenuation correction, standard error: SE,  All p-values < 10^–5^

### ScanOnlyDB: GLM with age, gender as an independent variable, and age-gender interaction

**Fig. 2:**
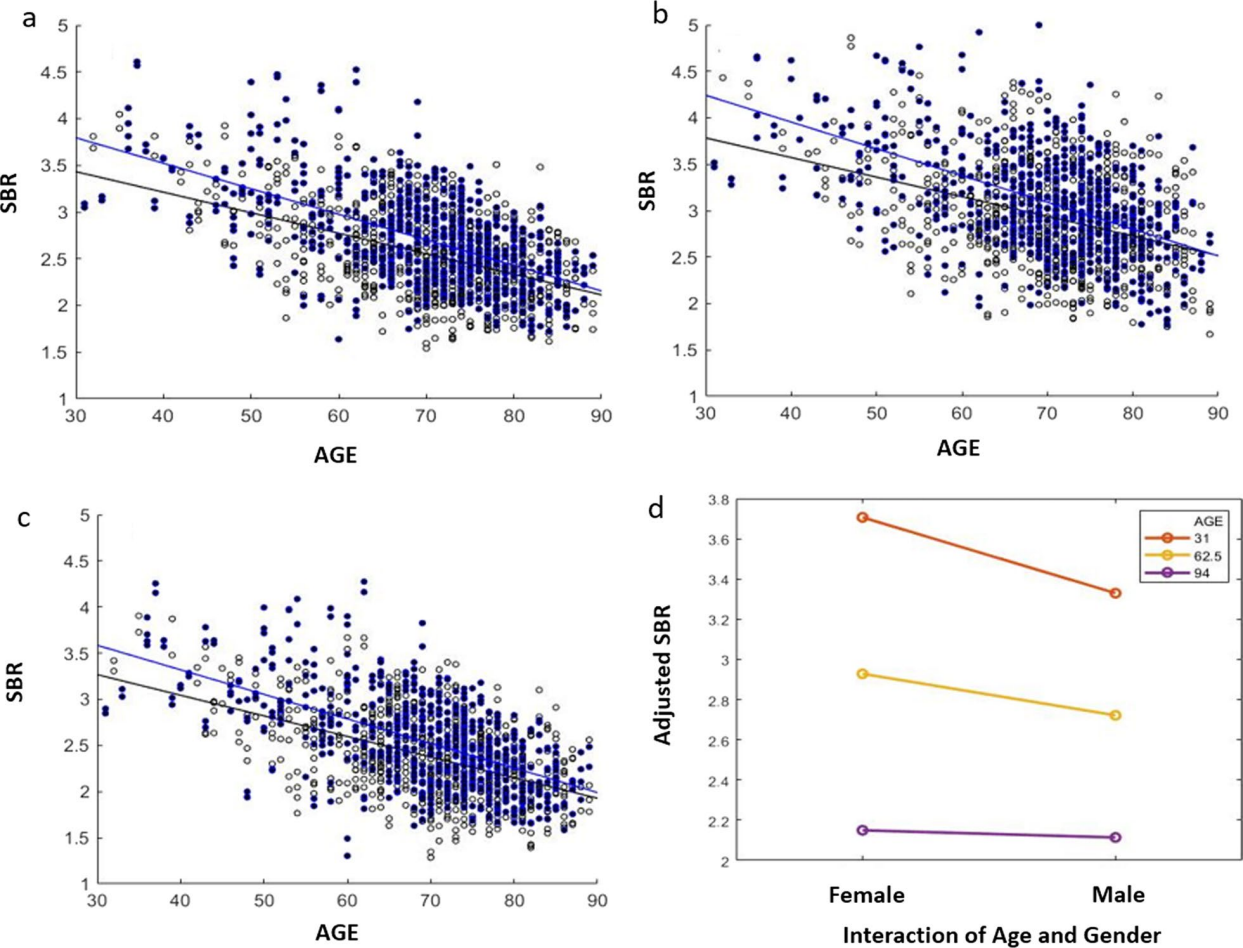
Scatterplots of ScanOnlyDB ^123^I FP-CIT SBR (Chang AC) values as a function of age for both male (open circle) and female (blue circle) for the three VOIs: Striatum (**a**), Caudate (**b**), and Putamen (**c**). Data relevant to each gender were independently fitted by linear regression lines (solid black line for males and solid blue line for females) and superimposed on each respective scatterplot. Age-gender interaction of striatal SBR for a mean age of 31 years (orange), 62.5 years (yellow), and 94 years (purple) is shown in (**d**)

The relationship between age and SBR in men and women is presented in Fig. 2 for the striatum (Fig. 2a), caudate (Fig. 2b), and putamen (Fig. 2c). The *R*^2^ values of the GLM were 0.33, 0.24, and 0.33 for CT AC and 0.31, 0.23, and 0.32 for Chang AC. Striatum Chang AC CoV was 15% after correction for mean age, whereas the SD was 0.40. Table 3 details the estimates of the GLM analysis for each variable. There was an age-gender interaction. For example, for Chang AC, estimates for interaction parameters per year were − 0.027, − 0.029, and − 0.027 (women) and − 0.022, − 0.021, and − 0.023 (men) for the striatum, the caudate, and the putamen, respectively. We computed adjusted SBR values according to age and gender at three different representative ages, 31 years, 62.5 years, and 94 years (Fig. 2d). At 31 years, female SBR values were 8.8% higher than male SBR values. This difference decreased with age (6.4% at 62.5 years and 0.9% at 94 years).

**Table 3:** Estimates of the general linear model analysis of SBR from the database ScanOnlyDB

Volume of interest	Parameter	SBR Chang AC		SBR CT AC	
		Estimate ± SE	*P*-values	Estimate ± SE	*P*-values
Striatum	Intercept	4.62 ± 0.120	*	3.71 ± 0.090	*
	Slope (age)	− 0.027 ± 0.001	*	− 0.023 ± 0.011	*
	Intercept (male)	− 0.52 ± 0.15	3.10^–4^	− 0.58 ± 0.12	*
	Slope (age*gender)	0.005 ± 0.002	9.10^–3^	0.006 ± 0.002	2.10^–4^
Caudate	Intercept	5.1 ± 0.130	*	4.3 ± 0.100	*
	Slope (age)	− 0.029 ± 0.001	*	− 0.023 ± 0.001	*
	Intercept (male)	− 0.69 ± 0.183	1.10^–4^	− 0.75 ± 0.147	*
	Slope (age*gender)	0.008 ± 0.003	3.10^–3^	0.008 ± 0.002	6.10^–5^
Putamen	Intercept	4.38 ± 0.098	*	3.696 ± 0.078	*
	Slope (age)	− 0.027 ± 0.001	*	− 0.022 ± 0.001	*
	Intercept (male)	− 0.45 ± 0.142	1.10^–3^	− 0.498 ± 0.113	1.10^–5^
	Slope (age*gender)	0.004 ± 0.002	3.10^–2^	0.005 ± 0.002	2.10^–3^

General linear model estimates of Specific Binding Ratios (SBR) from the database composed of visually normal scans (suspected Parkinsonism and no diagnostic follow-up, ScanOnlyDB) as a function of age, gender, and age-gender interaction (for the ‘male’ effect) for both attenuation correction methods: Chang and CT.

Chang AC: numerical method for attenuation correction, CT AC: computed tomography-based attenuation correction, standard error: SE, * p-values < 10^–5^

### HvDB: GLM analysis with age and gender as independent variables as well as age with gender interaction

For the HvDB, the GLM (*R*^2^ = 0.30) showed a covariation of SBR with age (estimate ± SE:− 0.026 ± 0.0032, *P* < 10^–5^). However, no significant covariation with gender (*P* = 0.4), nor age-gender interaction (*P* = 0.34) was found. The mean ± SD decrease in striatal SBR per decade was − 5.92 ± 0.24%. The linear regression equation of SBR_putamen_ to SBR_caudate_ ratio was estimate ± SE: 0.86 ± 0.03 + 0.0005*age (± 0.0006) but without a significant covariation with age (*P* = 0.39). After correction for mean age, the CoV for striatal SBR was 15% and the SD was 0.42.

### NoDG5YearsDB: GLM analysis with age and gender as independent variables and age-gender interaction

For the NoDG5YearsDB, the model (*R*^2^= 0.35) showed a covariation of SBR (estimate ± SE): with age − 0.022 ± 0.002; *P* < 10^–5^ and gender − 0.5 ± 0.175; *P* = 4*10^–3^). However, there was no significant age-gender interaction (*P* = 0.14). The mean ± SD change in striatal SBR per decade was − 4.65 ± 0.22%. After correction for mean age, the CoV for striatal SBR was 16% and the SD was 0.38.

### Comparison with ENC-DAT and SiemensDB

The *R*^2^ of the linear regressions of SBR with age were compared between the ScanOnlyDB, the HvDB, the NoDG5YearsDB, the ENC-DAT databases using BRASS analysis of uncalibrated ACSC data [6]and the SiemensDB [12](coefficients published for each VOI and gender in Table 4). No significant difference was found.

**Table 4:** SBR estimated parameters from ScanOnlyDB and those from the ENC-DAT and the SiemensDB

	Men				Women			
	ScanOnlyDB-Chang AC	ScanOnlyDB-CT AC	SiemensDB ACSC	Uncalibrated ACSC ENC-DAT	ScanOnlyDB-Chang AC	ScanOnlyDB-CT AC	SiemensDB ACSC	Uncalibrated ACSC ENCDAT
Striatum								
Slope, SE	− 0.022 ± 0.001	− 0.017 ± 0.001	− 0.015 ± 0.004	− 0.015 ± 0.006	− 0.027 ± 0.001	− 0.023 ± 0.001	− 0.018 ± 0.006	− 0.018 ± 0.007
* R*^2^	0.24	0.22	0.32	0.28	0.34	0.35	0.3	0.31
Caudate								
Slope, SE	− 0.021 ± 0.002	− 0.015 ± 0.001	− 0.016 ± 0.006	− 0.016 ± 0.006	− 0.029 ± 0.002	− 0.023 ± 0.001	− 0.017 ± 0.005	− 0.018 ± 0.007
* R*^2^	0.15	0.12	0.24	0.28	0.27	0.28	0.26	0.29
Putamen								
Slope	− 0.022 ± 0.001	− 0.017 ± 0.001	− 0.016 ± 0.006	− 0.016 ± 0.006	− 0.027 ± 0.001	− 0.022 ± 0.001	− 0.02 ± 0.005	− 0.017 ± 0.007
* R*^2^	0.25	0.24	0.38	0.28	0.34	0.35	0.33	0.29

Comparison of slopes (estimate ± standard error) and *R*^2^ parameters from ScanOnlyDB and those from the ENC-DAT study using BRASS analysis of uncalibrated ACSC data [6] and the SiemensDB [12]. ACSC, attenuation correction and scatter correction; ENC-DAT, European Normal Control Database of DaTscan™

### Combination of ScanOnlyDB and HvDB: GLM analysis with age and gender as independent variables and age-gender interaction

**Fig. 3:**
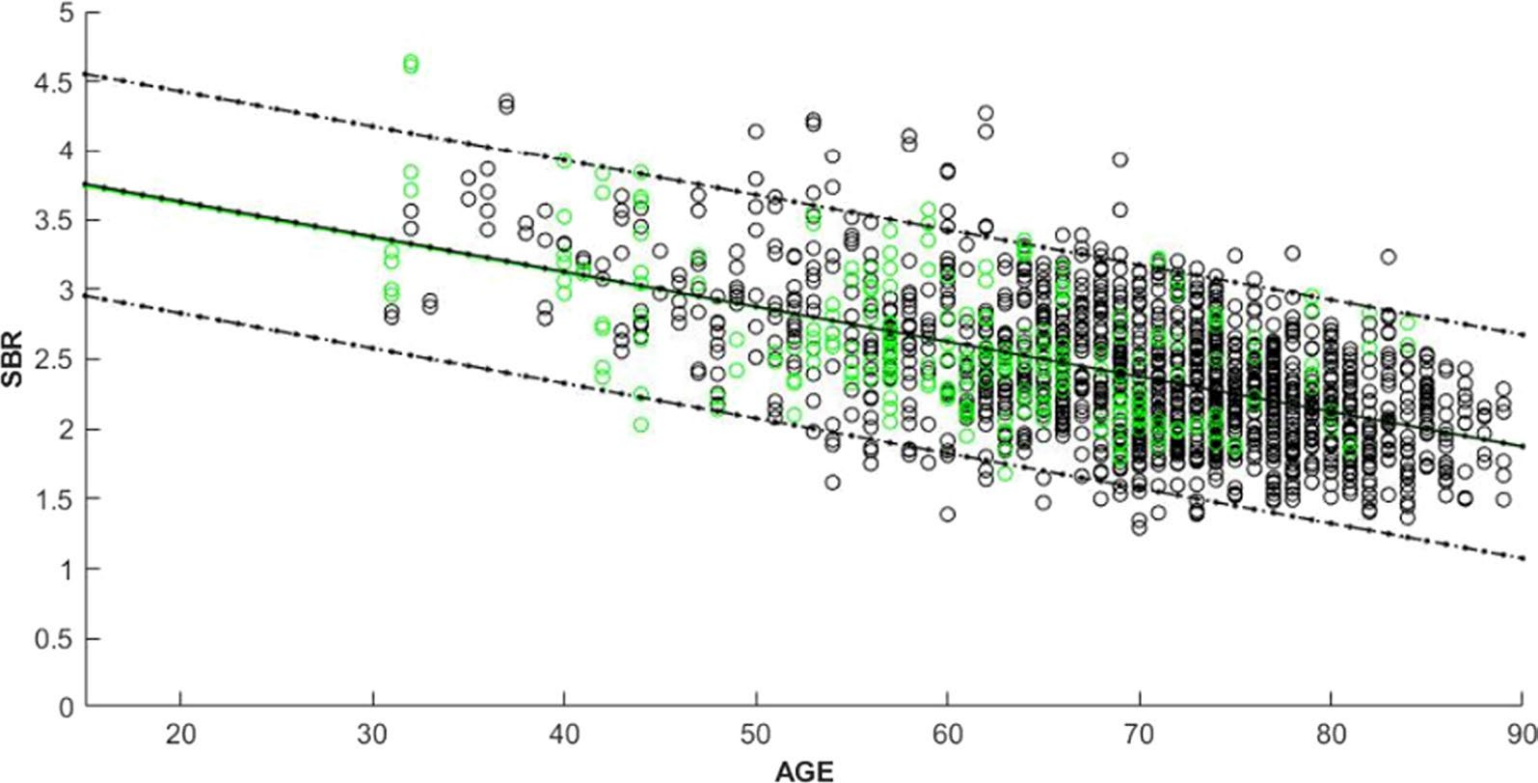
Scatterplot of harmonized ScanOnlyDB striatum VOI Specific Binding Ratio (SBR) values (*black open circles*) and HvDB SBR values (*green open circles*) as a function of age. Linear regression lines for age: ScanOnlyDB (*green solid line*) and ScanOnlyDB + NoDG5YearsDB ± 2 SD (*black solid and black dotted lines*) were superimposed

Figure 3 presents the SBR values of ScanOnlyDB and HvDB as a function of age after harmonization [SBR ScanOnlyDB harmonized = SBR ScanOnlyDB − 0.25]. The GLM analysis (*R*^2^= 0.27) showed a significant covariation with age (*P* < 10^–5^) and gender (*P* = 6.10^–3^). The slope ± SE was − 0.026 ± 0.001 and the intercept ± SE (male) was − 0.345 ± 0.13. There was no age-gender interaction (*P* = 0.08). The database factor was not significant (*P* = 0.76). After correction for mean age, the CoV for striatal SBR was 15% and the SD was 0.42.

### Combination of ScanOnlyDB and NoDG5YearsDB: GLM with age and gender as independent variables and age-gender interaction

**Fig. 4:**
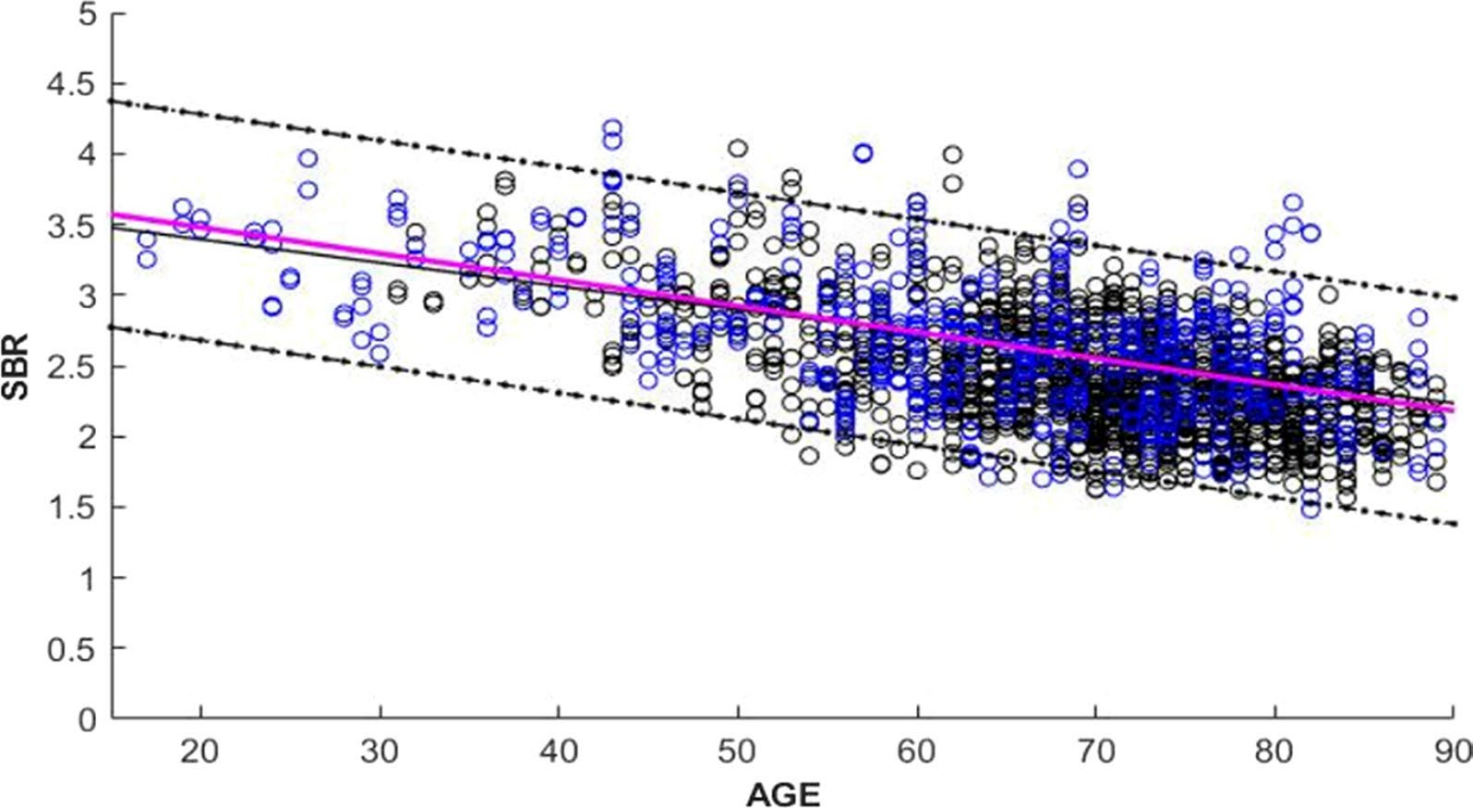
Scatterplot of harmonized ScanOnlyDB striatum VOI Specific Binding Ratios (SBR; *black open circles*) and NoDG5YearsDB (*blue open circles*). Linear regression lines for age: ScanOnlyDB (*magenta solid line*) and ScanOnlyDB + NoDG5YearsDB ± 2 SD (*black solid and black dotted lines*)

Figure 4 presents the SBR values of the harmonized SBR ScanOnlyDB harmonized = SBR ScanOnlyDB + 0.33] as a function of age. The GLM analysis (*R*^2^= 0.35) showed a significant covariation with age (*P* < 10^–5^) and gender (*P* < 10^–5^). The slope ± SE was − 0.021 ± 0.0009 and the intercept ± SE (male) was − 0.577 ± 0.09. The age-gender interaction slope ± SE was 0.0057 ± 0.001 (*P* = 6.10^–6^). The database factor was not significant (*P* = 0.9). After correction for mean age, the CoV for striatal SBR was 15% and the SD was 0.39.

## Discussion

In this study, we observed that the SBR values of normal scans from the ScanOnlyDB, derived from routine clinical activity, depended on age and gender, similarly to the SBR values of Hv from academic studies. SBR dispersion was not different (similar CoV and *R*^2^), thereby not providing an argument for an additional variance of the ScanOnlyDB database. These results were strengthened after combination of the databases, which constitutes an additional argument in favor of their non-difference. Furthermore, the SBR threshold (− 2SD) for age used to define an examination as abnormal did not differ between databases after post-processing harmonization.

The variations (decrease) in SBR values with age was comparable between the ScanOnlyDB and the SiemensDB [12], ENC-DAT [6], and data from histopathological studies [18, 19] Mean posterior putamen SBR values were lower than putamen SBR values and decreased at the same rate. The mean putamen and posterior-putamen SBR values were similar to those previously published [12]. These results are not consistent with those of a recent study [14] showing that the mean SBR values of Hv (*n* = 48) and of patients without dopaminergic degeneration (*n* = 70) were similar for all VOIs except for the posterior putamen VOI for which values were 8% higher for the patients. As suggested by the authors, this difference could be explained by a selection bias, thereby highlighting the importance of a large sample size.

The GLM analysis of the ScanOnlyDB found a negative linear regression between SBR and gender for the three VOIs, which is consistent with the results of the ENC-DAT, of the Japanese database, and of the SiemensDB study [6, 7, 12]. We did not find any interaction between age and gender for the HvDB database: this is possibly related to the use of different cameras and protocols without calibration [ causing an instrumental variance that masked this moderate effect. Gender discrimination was not shown to be necessary in order to differentiate a patient with Parkinson disease from a Hv [20] and DaTQUANT™ (GE Healthcare, Chicago, IL, USA), which ensures a semi-quantitative analysis without considering gender, is consistent with a routine clinical use for this diagnosis. This is also true for Siemens database [12]. However, a recent study has found the benefit of information on gender in reducing the risk of error for disease-free patients (particularly elderly men) and in distinguishing subjects with possible prodromal disease [21]. A database containing gender information could therefore present an additional useful resource in routine clinical practice.

The GLM analysis found an age with gender interaction for all VOIs investigated for the ScanOnlyDB. The more rapid decrease of SBR values for women with age (age-gender interaction) has regularly been described in published studies but using graphical analysis only, particularly for young subjects. This age-gender interaction was also found for Hv [7, 22] and for Flash3D AC CT Siemens DB [12], but not for ENC-DAT, nor HvDB. As the variance of this interaction was low compared to the total variance, a large sample was necessary to reveal it. This is one of the strengths of the ScanOnlyDB whose simple inclusion criterion allowed the inclusion of 764 patients, and thus the identification of these physiological dependencies.

The combined analysis of the harmonized ScanOnlyDB and NoDG5YearsDB (1001 normal scintigraphies) provided a high statistical power and therefore analytical robustness. The analysis did not find a significant difference between these two independent databases (built with different equipment, protocols, age distribution, and selection of diagnoses) and allowed the definition of reference values for the physiological parameters.

ScanOnlyDB presented dispersion parameters (notably CoV factor after correction for age) similar to those previously published [9, 23]. The *R*^2^ of the GLM models for the ScanOnlyDB explained up to 33% of the overall variance, which was similar to the ENC-DAT [23], the Japanese database [7], and the Siemens database [12]. Although part of the overall variance was related to age and gender, a large proportion of the overall variance remained unexplained (65–70%). This is probably because of the cross-sectional nature of studies, which do not take inter-individual variability into account, and which may also explain why the SD of the ScanOnlyDB and of the reference databases were similar. In addition, the − 2SD value used to define age-matched pathologic SBR values was not different after harmonization and combination of databases. It is of note that processing NoDG5yearsDB with DaTQUANT™ (GE Healthcare, Chicago, IL, USA) instead of the in-house methodology would increase homogeneity but SBR differences due to different acquisition pipelines would still be present, requiring harmonization. As the results presented herein were consistent even with pipelines using different processing methods based on VOI semi-quantitation the results are generalizable.

The ScanOnlyDB might include data from patients presenting neurodegenerative damage onset, more specifically global moderate symmetrical damage, which is difficult to demonstrate by visual analysis, but the SBR values were within the 2SD interval that defines normality. Severe global symmetrical damage can be detect as the images have a higher non-specific binding area relative enhancement (image intensity is scaled on the highest voxel, usually in the caudate area).

This is a limitation of the examination, but it did not translate into statistical difference between databases. If the diagnosis of dopaminergic degeneration was to be confirmed at follow-up, these patients would be qualified as “Scan Without Evidence of Dopaminergic Deficit” (SWEDD), but according to a recent review [24], their proportion was estimated only to be about 2%, which is similar to the 2.5% alpha risk (− 2SD).

## Conclusion

The distribution of the SBR values of visually normal examinations of patients with suspected Parkinsonism was not different from those of Hv or patients without dopaminergic degeneration. The large sample size of the databases analyzed confirmed the physiological dependencies published by large academic studies with Hv. The lower SBR threshold for age (− 2SD), used to define an examination as degenerative, was not different from the reference databases. The ScanOnlyDB database, containing information on gender, can be proposed to users of CZT gamma cameras with the same protocol. The results presented herein enable the creation of a reference database from routine visually normal clinical scans by expert nuclear medicine physicians. The proposed post-reconstruction harmonization method can also facilitate the use of semi-quantitative analysis across centers or cameras. This should favor the use of semi-quantitative analysis, in addition to visual analysis, for a more objective and standardized diagnosis.

## Supplementary Information

**Additional file 1**: ScanOnlyDB and NoDG5YearsDB diagnoses information and patient preparation.

**Additional file 2**: In-house SBR methodology.

**Additional file 3**: Description of NoDG5Years and Hv databases.

**Additional file 4**: Statistical analysis.

**Additional file 5**: SBR harmonization.

## Data Availability

The ScanOnlyDB datasets used and/or analyzed during the present study are available from the corresponding author upon reasonable request.

## Abbreviations

SPECT: Single-photon emission computerized tomography
GLM: General Liner Model
SBR: Specific Binding Ratio
CT: Computed tomography
CoV: Coefficient of Variation
AC: Attenuation correction
SC: Scatter Correction
CZT: Cadmium Zinc Tellure
SWEDD: Scan Without Evidence of Dopaminergic Deficit

## References

[1] Morbelli S, Esposito G, Arbizu J, Barthel H, Boellaard R, Bohnen NI (2020). EANM practice guideline/SNMMI procedure standard for dopaminergic imaging in Parkinsonian syndromes 1.0. Eur J Nucl Med Mol Imaging.

[2] Catafau AM, Tolosa E (2004). Impact of dopamine transporter SPECT using 123I-Ioflupane on diagnosis and management of patients with clinically uncertain Parkinsonian syndromes. Mov Disord Off J Mov Disord Soc.

[3] Papathanasiou N, Rondogianni P, Chroni P, Themistocleous M, Boviatsis E, Pedeli X (2012). Interobserver variability, and visual and quantitative parameters of (123)I-FP-CIT SPECT (DaTSCAN) studies. Ann Nucl Med.

[4] Thobois S, Prange S, Scheiber C, Broussolle E (2019). What a neurologist should know about PET and SPECT functional imaging for parkinsonism: a practical perspective. Highlights XXIII Lyon World Congr Park Dis Relat Disord [Internet]..

[5] Oliveira FPM, Walker Z, Walker RWH, Attems J, Castanheira JC, Silva Â (2021). (123)I-FP-CIT SPECT in dementia with Lewy bodies, Parkinson’s disease and Alzheimer’s disease: a new quantitative analysis of autopsy confirmed cases. J Neurol Neurosurg Psychiatry.

[6] Varrone A, Dickson JC, Tossici-Bolt L, Sera T, Asenbaum S, Booij J (2013). European multicentre database of healthy controls for [123I]FP-CIT SPECT (ENC-DAT): age-related effects, gender differences and evaluation of different methods of analysis. Eur J Nucl Med Mol Imaging.

[7] Matsuda H, Murata M, Mukai Y, Sako K, Ono H, Toyama H (2018). Japanese multicenter database of healthy controls for [(123)I]FP-CIT SPECT. Eur J Nucl Med Mol Imaging.

[8] The Parkinson Progression Marker Initiative (PPMI). Prog Neurobiol. 2011; 95:629–35. 10.1016/j.pneurobio.2011.09.005 PMC9014725 21930184

[9] Tossici-Bolt L, Dickson JC, Sera T, de Nijs R, Bagnara MC, Jonsson C (2011). Calibration of gamma camera systems for a multicentre European 123I-FP-CIT SPECT normal database. Eur J Nucl Med Mol Imaging [Internet]..

[10] Buchert R, Hutton C, Lange C, Hoppe P, Makowski M, Bamousa T (2016). Semiquantitative slab view display for visual evaluation of 123I-FP-CIT SPECT. Nucl Med Commun.

[11] Nicastro N, Garibotto V, Poncet A, Badoud S, Burkhard PR (2016). Establishing on-site reference values for (123)I-FP-CIT SPECT (DaTSCAN®) using a cohort of individuals with non-degenerative conditions. Mol Imaging Biol.

[12] Fahmi R, Platsch G, Sadr AB, Gouttard S, Thobois S, Zuehlsdorff S (2020). Single-site (123)I-FP-CIT reference values from individuals with non-degenerative parkinsonism-comparison with values from healthy volunteers. Eur J Hybrid Imaging.

[13] Lapa C, Spehl TS, Brumberg J, Isaias IU, Schlögl S, Lassmann M (2015). Influence of CT-based attenuation correction on dopamine transporter SPECT with [(123)I]FP-CIT. Am J Nucl Med Mol Imaging.

[14] Honkanen EA, Eklund M, Nuuttila S, Noponen T, Jaakkola E, Mäkinen E (2021). Dopamine transporter binding in symptomatic controls and healthy volunteers: considerations for neuroimaging trials. NeuroImage Clin.

[15] Chang L-T (1978). A method for attenuation correction in radionuclide computed tomography. IEEE Trans Nucl Sci.

[16] Cohen P, West SG, Aiken LS (2014). Applied multiple regression/correlation analysis for the behavioral sciences.

[17] Nobili F, Naseri M, De Carli F, Asenbaum S, Booij J, Darcourt J (2013). Automatic semi-quantification of [123I]FP-CIT SPECT scans in healthy volunteers using BasGan version 2: results from the ENC-DAT database. Eur J Nucl Med Mol Imaging.

[18] Fearnley JM, Lees AJ (1991). Ageing and Parkinson’s disease: substantia nigra regional selectivity. Brain J Neurol.

[19] Gibb WR, Lees AJ (1991). Anatomy, pigmentation, ventral and dorsal subpopulations of the substantia nigra, and differential cell death in Parkinson’s disease. J Neurol Neurosurg Psychiatry.

[20] Schmitz-Steinkrüger H, Lange C, Apostolova I, Mathies FL, Frings L, Klutmann S (2021). Impact of age and sex correction on the diagnostic performance of dopamine transporter SPECT. Eur J Nucl Med Mol Imaging.

[21] Postuma RB, Berg D (2019). Prodromal Parkinson’s disease: the decade past, the decade to come. Mov Disord [Internet].

[22] Lavalaye J, Booij J, Reneman L, Habraken JB, van Royen EA (2000). Effect of age and gender on dopamine transporter imaging with [123I]FP-CIT SPET in healthy volunteers. Eur J Nucl Med.

[23] Buchert R, Kluge A, Tossici-Bolt L, Dickson J, Bronzel M, Lange C (2016). Reduction in camera-specific variability in [(123)I]FP-CIT SPECT outcome measures by image reconstruction optimized for multisite settings: impact on age-dependence of the specific binding ratio in the ENC-DAT database of healthy controls. Eur J Nucl Med Mol Imaging.

[24] Roberta B, Paolo B, Massimo F, Roberto E (2022). Unexpected ((123)I)FP-CIT SPECT findings: SWIDD, SWEDD and all DAT. J Neurol.

