# Supplementary material for: Semi-quantitative analysis of visually normal 123I-FP-CIT across three large databases revealed no difference between control and patients
Source: EJNMMI Res. 2023 Apr 28;13:37. doi: 10.1186/s13550-023-00983-6 (PMC10147889; doi:10.1186/s13550-023-00983-6)
Supplement: Supplementary file 1 — Additional file 1: ScanOnlyDB and NoDG5YearsDB diagnoses information and patient preparation. [file 13550_2023_983_MOESM1_ESM.docx]

**Supplementary Table 1** : ScanOnlyDB and NoDG5yearsDB diagnoses information

| Diagnosis | Clinically suspected diagnoses before ^123^I FP-CIT  in the ScanOnlyDB, % | n | Confirmed diagnoses in the NoDG5YearsDB, % | n |
| --- | --- | --- | --- | --- |
|  |  |  |  |  |
|  |  |  |  |  |
| Essential tremor | 23.7 | 181 | 27 | 64 |
| Drug-induced parkinsonism | 23.2 | 177 | 22.8 | 54 |
| Dementia without dopaminergic degeneration (Alzheimer’s disease [AD] n=34, or frontotemporal dementia n=17, mixed Alzheimer's disease & Lewy body disease n=54 | 12.4 | 95 | 10.5 | 25 |
| Attention Deficit Hyperactivity disorder | none | none | 8.5 | 20 |
| Miscellaneous or mixed (rheumatologic disorders, Cerebral RX therapy, Neurosurgery) | 8.8 | 67 | 10.1 | 24 |
| Vascular parkinsonism without dopaminergic degeneration | 5.2 | 40 | 6.1 | 14 |
| Psychiatric disorders, psychogenic parkinsonism, or movement disorders | 6.8 | 52 | 6.3 | 15 |
| Normal pressure hydrocephalus | 1.8 | 14 | 2.5 | 6 |
| Dystonia | 1.7 | 13 | 1.3 | 3 |
| Restless-leg syndrome | 0.9 | 7 | 1.3 | 3 |
| Amyotrophic lateral sclerosis | 0.1 | 1 | 0.8 | 2 |
| Epilepsy | 0.3 | 2 | 0.8 | 2 |
| Genetic form of Parkinson’s disease | 0.1 | 1 | 0.8 | 2 |
| Cerebellar ataxia | 1.4 | 11 | 0.5 | 1 |
| Undetermined atypical parkinsonism, n=103 including n=47, possible Multiple System Atrophy=30, possible Supra nuclear palsy=8, possible cortico-basal degeneration=9 | 13.5 | 103 | excluded | excluded |
| Total | 100 | 764 | 100 | 237 |

**Supplementary table 1.** Clinically suspected diagnosis for the ScanOnlyDB and after follow-up for NoDG5yearsDB

**ScanOnlyDB Patient preparation**

To avoid interactions, all drugs that could alter the uptake of ^123^I-FP-CIT were discontinued at least a week before the examination. Other medications in general, and dopaminergic agents in particular, whenever used were not discontinued. All patients were administered intravenously a mean ± standard deviation (range) dose of ^123^I-FP-CIT of 146±24 (110-185) MBq.
