# Supplementary material for: Semi-quantitative analysis of visually normal 123I-FP-CIT across three large databases revealed no difference between control and patients
Source: EJNMMI Res. 2023 Apr 28;13:37. doi: 10.1186/s13550-023-00983-6 (PMC10147889; doi:10.1186/s13550-023-00983-6)
Supplement: Supplementary file 2 — Additional file 2: In-house SBR methodology. [file 13550_2023_983_MOESM2_ESM.docx]

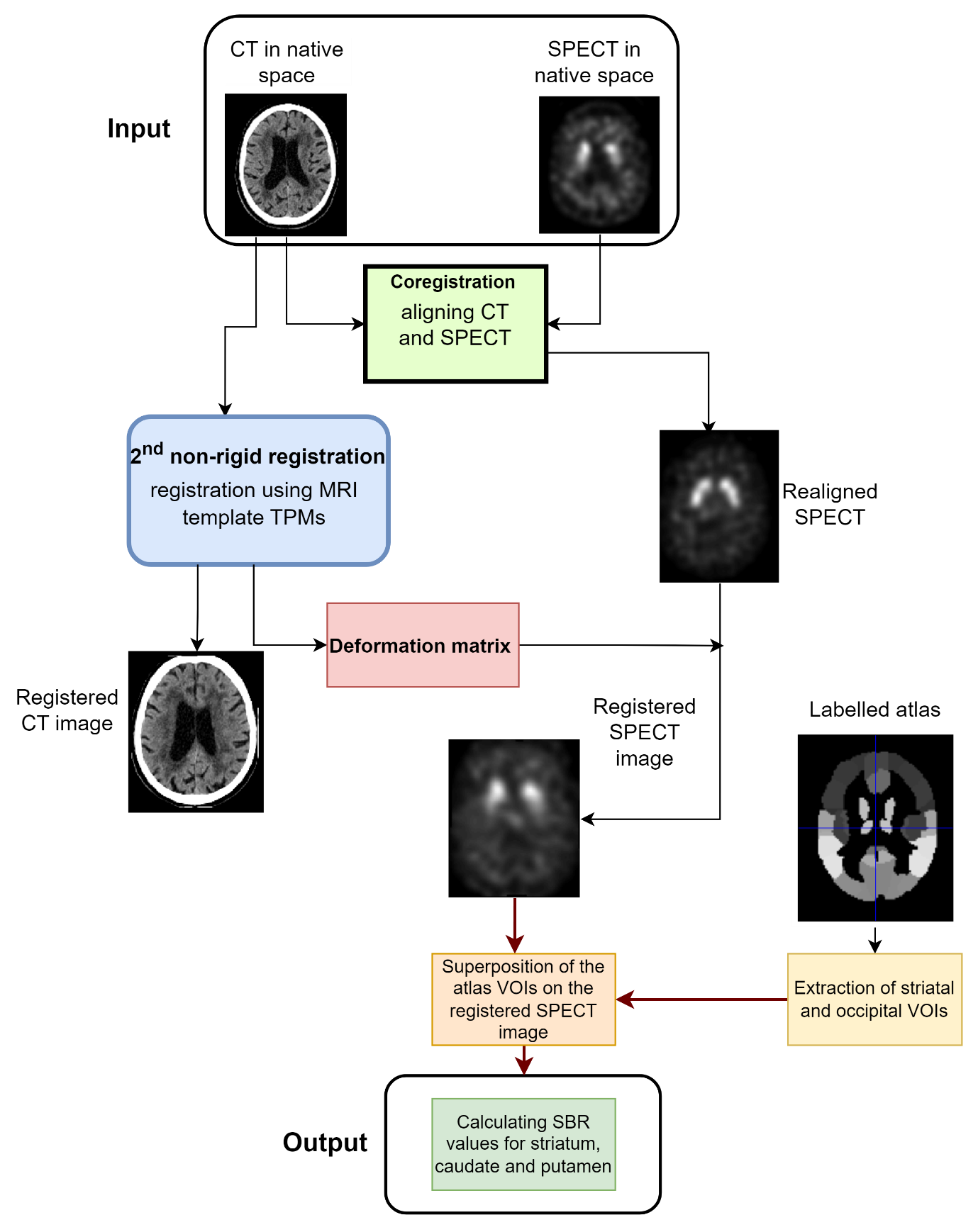
Supplementary Material 2: In-house SBR methodology

Fig. 1 Pipeline of our in-house software for semi-quantification of 123I FP-CIT images

The in house software for the semi-quantitative analysis of ^123^I FP-CIT SPECT images through CT-based volume-of-interest (VOI) in a standard anatomical space.

The aim is to spatially normalize the SPECT image to a common anatomical space to overcome registration failure in case of low tracer uptake. Firstly, CT and SPECT images of the same patient are rigidly co-registered using the CT as reference. Then, the CT image is non-rigidly registered to a common template using SPM12 (Wellcome Centre for Human Neuroimaging, UCL Queen Square Institute of Neurology, London, UK) unified framework for segmentation and registration. The deformation matrix is then applied to the SPECT image. Next, a labelled atlas (MNI V4) in the common space is used to extract voxel intensity in the striatal and occipital VOIs. Specific binding ratios (SBR) are calculated according to the following formula using values from the different ROIs: SBR= (Striatum-Occipital) /Occipital. Compared to other commercial semi-quantification software, the in-house software allows further tuning and more flexible image processing experience. The software is fully-automated. This software has been used to calculate SBRs from NoDG5yearsDB and the results have been compared with those from Siemens Syngo Via software ^TM^ (Siemens Medical Solutions USA, Inc) on the same data set [1] after harmonization.

Reference

1. Fahmi R, Platsch G, Sadr AB, Gouttard S, Thobois S, Zuehlsdorff S, et al. Single-site (123)I-FP-CIT reference values from individuals with non-degenerative parkinsonism-comparison with values from healthy volunteers. Eur J Hybrid Imaging. 2020;4:5.
