# Supplementary material for: Semi-quantitative analysis of visually normal 123I-FP-CIT across three large databases revealed no difference between control and patients
Source: EJNMMI Res. 2023 Apr 28;13:37. doi: 10.1186/s13550-023-00983-6 (PMC10147889; doi:10.1186/s13550-023-00983-6)
Supplement: Supplementary file 3 — Additional file 3: Description of NoDG5Years and Hv databases. [file 13550_2023_983_MOESM3_ESM.docx]

**Supplemental Material 3:**

**Description of reference databases**

**NoDG5YearsDB** contains examinations that are normal on visual and semi-quantitative analysis. This heterogeneous series (Supplementary Table 1) was acquired in the Hospices Civils de Lyon on a SPECT-CT Symbia T2 camera (Siemens Medical Solutions USA, Inc) between January 2008 and December 2015, following EANM recommendations [1]. It was composed of 237 subjects, matched for age and gender: there were 120 (50.6%) men, 117 (49.4%) women, and their mean±SD (range) age was 62.2±15.7 (16–88) years. The particular feature of this cohort is the inclusion of young adults with attention deficit hyperactivity disorders; patients with suspected atypical parkinsonism were excluded. After a mean±SD follow-up duration of 4.8±1.3 years, the absence of dopaminergic neurodegenerative pathology was confirmed. The experimental protocol included scatter correction using the triple energy window method, a correction for the spatial response of the camera, and CT attenuation correction; these were integrated into the Flash3D software (OSEM3D with resolution recovery) with 10 iterations and 8 subsets). Semi-quantification was carried out using software developed in the department (Supplementary Material 2). In addition, raw data were processed for a second time by Siemens to construct a database of reference values, SiemensDB [2]

**HvBD** is the database supplied with DaTQUANT^TM^ (GE Healthcare, Chicago, IL, USA). It is composed of examinations normal on visual analysis of healthy volunteers derived from data from the Parkinson’s Progression Markers Initiative cohort database [3]. It contains 118 subjects. The mean±SD (range) age of subjects included in the cohort was 60.3±13 (31-84) years, there were 73 (61%) men and 45 (39%) women. Acquisitions were made in different centers using different gamma cameras with NaI detectors. The photopeak energy window was 159 keV (±10%). Image reconstruction and SBR measurement were carried out on a Xeleris 4.0 workstation according to the protocol proposed by DaTQUANT^TM^ (GE Healthcare, Chicago, IL, USA): 2D-OSEM (2 iterations, 10 subsets, Butterworth filter: 0.6, 10) for the volumes of interest (VOI) striatum, caudate, putamen. Attenuation correction was carried out according to the Chang method.

References

1. Morbelli S, Esposito G, Arbizu J, Barthel H, Boellaard R, Bohnen NI, et al. EANM practice guideline/SNMMI procedure standard for dopaminergic imaging in Parkinsonian syndromes 1.0. Eur J Nucl Med Mol Imaging. 2020;47:1885–912.

2. Fahmi R, Platsch G, Sadr AB, Gouttard S, Thobois S, Zuehlsdorff S, et al. Single-site (123)I-FP-CIT reference values from individuals with non-degenerative parkinsonism-comparison with values from healthy volunteers. Eur J Hybrid Imaging. 2020;4:5.

3. Marek K. The Parkinson Progression Marker Initiative (PPMI). Prog Neurobiol. 2011;95:629–35.
