# Supplementary material for: Semi-quantitative analysis of visually normal 123I-FP-CIT across three large databases revealed no difference between control and patients
Source: EJNMMI Res. 2023 Apr 28;13:37. doi: 10.1186/s13550-023-00983-6 (PMC10147889; doi:10.1186/s13550-023-00983-6)
Supplement: Supplementary file 4 — Additional file 4: Statistical analysis. [file 13550_2023_983_MOESM4_ESM.docx]

**Supplementary Material 4: Statistical analysis**

The analyses were conducted while taking into account both striata, both caudate nuclei, and both putamina, including the posterior putamen, as independent variables. The analysis was conducted using a GLM: the SBR were the dependent variables and the independent variables tested were laterality, age, gender, and their interaction. Subjects over 75 years of age presenting values higher than +3 SD were excluded from the GLM analysis in light of a probable overestimation of SBR because of occipital atrophy and/or high dopaminergic reserve. The normal distribution of residuals was checked visually via a Normal Q-Q plot and an analysis of variance (ANOVA) was performed. The relevance of each model was tested using the root mean squared error*,* the coefficient of determination R^2^, and the F statistic vs. constant model, its significance p, and the Bayesian information criterion (BIC).
