# Supplementary material for: Semi-quantitative analysis of visually normal 123I-FP-CIT across three large databases revealed no difference between control and patients
Source: EJNMMI Res. 2023 Apr 28;13:37. doi: 10.1186/s13550-023-00983-6 (PMC10147889; doi:10.1186/s13550-023-00983-6)
Supplement: Supplementary file 5 — Additional file 5: SBR harmonization. [file 13550_2023_983_MOESM5_ESM.docx]

**Supplemental Material 5**

**Post-reconstruction harmonization: comparing SBR striatal reference values from different origins**

Rationale: it is well known that many parameters affect SBR values (gamma-camera type, acquisition settings, reconstruction algorithms, and parameters, attenuation and scatter correction and semi-quantitation programs). Data harmonization is usually addressed by calibration (test objects) followed by a single processing pipeline of the reconstructed raw data or calibrated raw data followed by a single reconstruction, correction, and semi-quantitation pipeline.

The aim was to demonstrate the similarity of reference values (known physiological dependencies as criteria) from routine clinical studies, but we had to deal with SBR obtained from different pipelines.

We postulate that the net effect of the pipeline on SBR can be divided into two categories: 1) addition/subtraction of a constant SBR, and 2) which depends on the SBR value.

We know from the literature [1] that SBR obtained after Chang attenuation correction (AC) and after CT AC are linearly related. This was confirmed by computing a linear regression between ScanOnlyDB CA AC and Scan OnlyDB Chang AC (SBR CT~1+SBR Ch: 0.03+0.83*SBR Ch, n=1330, R^2^=0.964, p=0.006). We therefore had to pair databases according to the attenuation correction method.

When we compared linear regression for each paired databases, the global effect was an offset (subtraction of a constant SBR).

The harmonization used SBR files processed with the same AC method. ScanOnlyDB SBRs were generated twice (CT AC and Chang AC). GLM analysis: SBR_striatal_ as the dependent variable and age as the independent variable

1) harmonization between ScanOnlyDB and NoDG5yearsDB

Use ScanOnlyDB CTAC to match NoDG5yearsDB (CT AC)

SBR _striatum ScanOnlyDB CTAC_ =intercept _striatum ScanOnlyDB CTAC base1_ +slope _striatum ScanOnlyDB CTAC_  (year^-1^)

SBR _striatum NoDG5yearsDB_ =intercept _striatum NoDG5yearsDB CTAC_ +slope _striatum NoDG5yearsDB CTAC_ (year^-1^)

SBR _striatumNoDG5yDB harmonized_=SBR_striatumNoDG5yDB_*(intercept _striatum ScanOnlyDBCTAC_ /intercept _striatum NoDG5yearsDB CTAC_)

2) harmonization between ScanOnlyDB and HvDB

Use ScanOnlyDB Chang AC to match HvDB (Chang AC)

SBR _striatum ScanOnlyDB Chang AC_ =intercept _striatum ScanOnlyDB Chang AC_ +slope _striatum ScanOnlyDB Chang AC_ (year^-1^)

SBR _striatum HvDB_ =intercept _striatum HvDB_ +slope _striatum HvDB_ (year^-1^)

SBR _striatumHvDB harmonized_=SBR_striatum HvDB_*(intercept _striatum ScanOnlyDB Chang AC_ /intercept

Reference

1. Lapa C, Spehl TS, Brumberg J, Isaias IU, Schlögl S, Lassmann M, et al. Influence of CT-based attenuation correction on dopamine transporter SPECT with [(123)I]FP-CIT. Am J Nucl Med Mol Imaging. 2015;5:278–86.
